# Closing the loop: human-augmented, mechanistically enhanced AI for proactive management of drug–drug interactions

**DOI:** 10.3389/fphar.2026.1767646

**Published:** 2026-03-25

**Authors:** Marios Spanakis, Anthe De Pauw, Maja Brumer, Emmanouil K. Symvoulakis, Hans De Loof

**Affiliations:** 1 Department of Social Medicine, School of Medicine, University of Crete, Heraklion, Greece; 2 Community Pharmacists Association of Heraklion, Crete, Greece; 3 Laboratory of Physiopharmacology, University of Antwerp (UA), Antwerp, Belgium; 4 Clinical pharmacy, Antwerp University hospital, Edegem, Belgium; 5 Infla-Med Research Center of Excellence, University of Antwerp, Antwerp, Belgium

**Keywords:** adverse drug reactions, artificial intelligence, Drug–drug interactions, explainable AI, human-in-the-loop AI, modeling and simulation, pharmacovigilance, real-world data

## Abstract

Drug–drug interactions (DDIs) represent a major challenge in pharmaceutical research for ensuring safe and effective medication use in clinical practice. Pharmacological DDI assays generate data that underpin clinical guidelines, drug interaction checkers, and decision support systems. Although these approaches remain indispensable, contemporary clinical practice is far more complex, shaped by polypharmacy, multimorbidity, diverse phenotypes, and social determinants of health. Artificial intelligence (AI) offers opportunities to integrate molecular, pharmacokinetic, and pharmacodynamic knowledge with real-world observations, enabling more proactive and patient-centered approaches to DDI risk assessment. This perspective proposes a conceptual framework for transitioning from static, rule-based DDI tools toward human-augmented AI systems in which clinician feedback is embedded as an integral component of model learning and interpretation, rather than serving solely as *post hoc* validation. Through structured incorporation of clinical expertise, experimental pharmacology is continuously contextualized against real-world decision-making. The manuscript outlines an AI ecosystem that is ethically grounded, mechanistically informed, and enriched through pharmacovigilance data and systematic clinician input. By operationalizing human-in-the-loop learning as a core design principle, this framework establishes human-augmented AI as a foundational paradigm for future DDI research, drug development, and personalized medication safety.

## Introduction

Drug safety in healthcare has become increasingly complex, driven by the prevalence of polypharmacy, multimorbidity, as well as social and behavioral determinants on medication use (Mehta and Alexander, 2024; Marengoni and Onder, 2015). One of the major challenges is the mitigation of drug-drug interactions (DDIs) within therapeutic regimens to avoid complications and adverse drug reactions (ADRs). Drug research and development (R&D) has long relied on a continuum of experimental pharmacology and advanced modeling and simulation (M&S) approaches, including sophisticated physiologically based pharmacokinetic (PBPK) models, to quantitatively predict drug exposure and interaction risk in relation to safety, efficacy, biomarkers, and off-target toxicities (Pognan et al., 2023). These predictions are subsequently assessed through targeted *in vivo* studies and small-scale clinical trials (Lohy Das et al., 2025). Collectively, these methods remain indispensable, shaping regulatory decisions, informing the formulation of summaries of product characteristics (SmPCs), and supporting observational studies and clinical decision-support systems (CDSS) (Jamei, 2016; Dimakos and Douros, 2024; Scheife et al., 2015; Horn et al., 2011; Li et al., 2022). Yet, despite their value, they are inherently performed under controlled conditions and therefore do not always capture the multidimensional complexity encountered in everyday clinical practice (Weaver and Valentin, 2019). Real-world pharmacotherapy is embedded within complex exposure networks, where prescribed medications coexist with over-the-counter (OTC) products, dietary and herbal supplements, diet habits and lifestyle-associated factors, collectively influencing pharmacological effects through multiple biological and behavioral mechanisms (Qato et al., 2008; Agbabiaka et al., 2017; Moschny et al., 2021; Spanakis et al., 2022). Conventual pharmacological frameworks, however, tend to reduce this complexity to a binary assessment of whether two compounds interact and whether it is clinically significant. This simplification creates an evidence gap between mechanistic predictions and the actual risk landscape that unfolds in daily clinical practice. For example, a combination classified as “moderate” or “of caution” may become clinically important when present in a regimen involving several additional moderate-risk DDIs, frailty, phenotypic variation in drug clearance, or reduced adherence shaped by socioeconomic determinants (Hwang et al., 2023; Mallet et al., 2007; Bories et al., 2021; Rojas et al., 2023). Existing DDI checkers and CDSS often fail to account for cumulative risk, patient-specific vulnerabilities (e.g., organ dysfunction, age, comorbidities), issues related with fragmented care or social determinants of health (SDoH) that influence adherence, exposure patterns, and ADR susceptibility (Andersson et al.,2018; Pestka et al., 2020; Kontsioti et al., 2022; Van De Sijpe et al., 2022; Barankay, 2025; Bellanca et al., 2025).

In parallel, the last decade we witness a fruitful expansion of artificial intelligence (AI) applications aimed at predicting drug, disease, and nutrient interactions using databases such as DrugBank, PubChem, ClinPGx (former PharmGKB), NAR, *etc.* (Barbarino et al., 2018; Knox et al., 2024; Spanakis et al., 2025a; Rigden and Fernández, 2024). Machine learning (ML), deep learning (DL), graph neural networks (GNN), and knowledge graph-based models are used to infer DDI risks across mechanistic and phenotypic domains (Vo et al., 2022; Marzouk et al., 2025; Zhang et al., 2024). These methods integrate heterogeneous data such as molecular structures, networks, PK/PD features, and ADRs in order to find patterns that extend beyond traditional statistical approaches. Despite progress, key limitations persist. Many AI systems prioritize prediction performance over clinical transparency, depend on datasets that may lack balance or representativeness, and offer minimal explanation of their decision-making processes (Ennab and Mcheick, 2024). Their black-box nature raises concerns for risk-sensitive pharmacotherapy, underscoring the importance of explainable AI (XAI) to clarify which molecular, PK/PD, pharmacogenetic, or behavioral features drive patient-specific risk (Alizadehsani et al., 2024; Thalpage, 2023). Moreover, many AI-DDI systems remain partially validated in real-world settings, restricting their clinical utility, mirroring challenges observed when current large language models (LLMs) are used to answer general medication-related queries (Radha Krishnan et al., 2024; Mondal et al., 2025). Concurrently, the impending link of experimental pharmacology, mechanistic modeling (i.e., PBPK), and real-world evidence through AI offers an opportunity to develop more adaptive, patient-centered approaches to DDI assessment (Huang et al., 2025).

Achieving this transition requires a conceptual shift from static prediction tools toward dynamic systems that integrate mechanistic understanding with the realities of clinical care. In this Perspective, we outline a framework for AI-enabled DDI assessment, conceptualized as a human-augmented, mechanistically enhanced ecosystem that learns bidirectionally from experimental pharmacology and real-world clinical experience. Rather than optimizing isolated predictions, this approach emphasizes co-evolution between AI systems, clinical reasoning, pharmacovigilance signals, and patient-specific modifiers.

Human-in-the-loop AI, reinforcement learning from human feedback (RLHF), and reinforcement learning–based dynamic treatment regimens are increasingly applied in healthcare, particularly in precision medicine, critical care, and NLP-driven clinical decision support (Sassi et al., 2025; Israni et al., 2025; Frommeyer et al., 2025; Chen et al., 2024). Yet these approaches are often domain-agnostic or optimized for sequential treatment selection rather than for interpreting DDI-related clinical risk, where identical mechanistic premises may yield divergent outcomes across patients. In addition, current human-in-the-loop AI approaches require further clinical research to determine how effectively human oversight addresses uncertainty and error in complex clinical contexts (Wang et al., 2026). In either case, expert human input remains essential for resolving such complexity, particularly when model confidence, interpretability, and clinical relevance diverge (Idan et al., 2025; Frommeyer et al., 2025; Carone and Rotnitzky, 2026; Liu et al., 2024).

**FIGURE 1:**
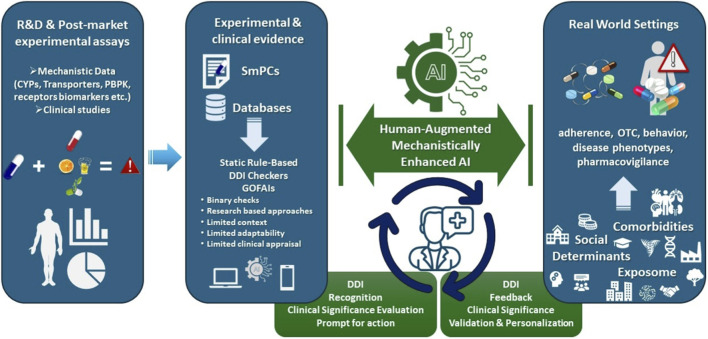
Conceptual framework for a human-augmented, mechanistically-enhanced AI (HAMEAI) ecosystem for advancing DDI prediction and management.

In this context, we position AI-enabled DDI assessment at the intersection of mechanistic pharmacology, PBPK modeling, multi-omics biomarkers, and social and behavioral determinants—domains that remain insufficiently integrated within current computational frameworks. This Perspective proposes a conceptual transition across three dimensions (Figure 1): (i) bridging the evidence gap between experimental knowledge and real-world clinical complexity; (ii) enabling bidirectional learning through structured clinician feedback; and (iii) advancing transparent, patient-centered, and mechanistically grounded AI systems.

## Reframing the evidence gap: from experimental knowledge to real-world complexity

The scientific foundations of DDIs research, both in drug R&D and post-marketing surveillance, are rooted in experimental pharmacology. Molecular targets, enzyme kinetics, transporter modulation, receptor affinities, and PK/PD profiles provide early evidence of interaction potential (Sudsakorn et al., 2020; Prueksaritanont et al., 2013). *In vitro* and *in silico* approaches enable the analysis of metabolic pathways and the prediction of modulating effects on enzymes such as Cytochrome P450 isoforms (e.g., CYP3A4, CYP2D6, CYP2C, *etc.*) or key transport systems (e.g., P-gp, OATP1B1/3, BCRP, *etc.*) with simulations of potential clinical outcomes (Foti, 2025). Integration of multiomics data (e.g., genomics, transcriptomics, proteomics, metabolomics) provide a systems-level perspective on complex biological network effects that are subsequently validated through *in vivo* and clinical studies (Zack et al., 2025; Alemu et al., 2025). These experimental layers are indispensable, forming the basis for interaction hypotheses, clinical trial simulations, and labeling recommendations in SmPCs. Nevertheless, translating mechanistic plausibility into real-world clinical significance remains challenging (Kadambi et al., 2025; ICH, 2025). Patients often use prescription medications along with OTC products or dietary supplements while lifestyle habits, environmental exposures, psychosocial stressors, and broader SDoH shape overall treatment outcome (Wilder et al., 2021; Emeny et al., 2022). Such factors can modify treatment-related behaviors, including adherence, systemic inflammation, metabolic capacity, and age-related organ dysfunction substantially modifying both the probability and impact of DDIs beyond what is captured in preclinical evaluation. As a result, DDI risk emerges not as a discrete pharmacological event but as a cumulative, context-dependent phenomenon shaped by interacting biological, behavioral, and social layers—analogous to other multidimensional biological processes that resist reductionist interpretation (Wright, 2022). A mechanistically predicted DDI may be clinically irrelevant in one patient yet highly significant in another (i.e., due to CYP polymorphisms). Similarly, polypharmacy patterns involving multiple moderate-risk DDIs may generate additive or synergistic PK or PD burdens capable of precipitating ADRs (Zhao et al., 2024; Malki and Pearson, 2019).

Traditional expert systems, DDI checkers and CDSS systems, representing forms of Good Old-Fashioned AI (GOFAI), remain constrained by static, rule-based architectures and that cannot accommodate this evolving complexity in pharmacotherapy (Kontsioti et al., 2022; Van De Sijpe et al., 2022; Papadopoulos et al., 2022; Spanakis et al., 2025b). These systems neither co-evolve with expanding scientific knowledge nor account for contextual modifiers such as pharmacogenetic variability, psychosocial stressors, exposome-related exposures, or dynamic phenotype shifts in hepatic or renal function. Furthermore, despite their contribution to computational pharmacology and biomedical research, many computational models focus on detecting or classifying DDIs rather than the evaluation of their clinical significance (Zirkle et al., 2023; Yin et al., 2023; Schwarz et al., 2021; Lee et al., 2019; Yang et al., 2023). Although pharmacological databases are rich in curated mechanistic data, they provide limited insight into how DDIs unfold under conditions (Abbas et al., 2025; Li et al., 2025; Lin et al., 2023; Li Z. et al., 2023). Similar concerns apply to the emerging use of LLMs in medication safety, where limitations in reliability and contextual understanding have been widely reported (Al Meslamani and Abou Hajal, 2025; Qi et al., 2025; Aydin et al., 2025; De Busser et al., 2024; Al-Ashwal et al., 2023; Del Rio-Bermudez et al., 2020).

These constraints contribute to a persistent mismatch between rapidly advancing mechanistic knowledge and its translation into actionable clinical guidance. Unlike pattern-recognition domains such as in medical image or electrocardiogram analysis, DDI assessment requires individualized interpretation, as the presence of a mechanistic interaction pathway does not guarantee clinical manifestation or relevance (Van De Sijpe et al., 2022; Villa Zapata et al., 2022). Bridging this gap requires reframing DDI evaluation as a multidimensional pharmacological process shaped by molecular, physiological, behavioral, environmental, and social determinants, each contributing distinct layers of DDI risk in everyday care (ICH, 2025; Dagenais et al., 2024; Woosley et al., 2021; Watch List: Artificial Intelligence in Health Care, 2025). To address these limitations, we advocate for a Human-Augmented, Mechanistically-Enhanced AI (HAMEAI) framework that integrates experimental pharmacology with real-world data and continuous clinician feedback. HAMEAI conceptualizes a DDI-specific learning ecosystem capable of informing drug development, regulatory risk assessment, pharmacovigilance, and individualized clinical decision-making (Figure 1), without introducing a new algorithmic class. Instead, clinician feedback is explicitly anchored to mechanistic pharmacology and real-world outcomes, enabling contextual interpretation of cumulative and patient-specific DDI risk.

## Building a bidirectional AI-Learning system: from top-down feedback to adaptive clinical systems

Bridging experimental evidence and clinical reality requires integration of bottom-up mechanistic data with top-down information reflecting real-world therapeutic experience. While recent frameworks have combined AI knowledge graphs and CDSS to enhance DDI prediction, clinician feedback is often treated as external validation or alert override (Huang et al., 2025). In contrast, HAMEAI conceptualizes clinician feedback as structured learning input, where confirmation, dismissal, or annotation of DDI alerts reflects context-aware interpretation of clinical significance rather than system error. This distinction enables adaptive recalibration of DDI risk in relation to mechanistic plausibility, cumulative exposure, and patient-specific modifiers.

Pharmacovigilance databases, electronic health records, clinical registries, pharmacoepidemiological studies, and medication review processes capture DDI patterns not anticipated by preclinical models (Ventola, 2018; Nagar et al., 2025; Price, 2023; Hauben, 2023). Pharmacogenetic and pharmacogenomic data further link genetic variation to enzyme activity, transporter function, receptor sensitivity, and phenotype expression, offering critical modifiers of individual susceptibility to DDIs (Zack et al., 2025). Together, these data sources reflect population heterogeneity, longitudinal physiological change, and comorbidity-related effects that are largely absent from controlled experimental conditions (Kadambi et al., 2025; Anagnostopoulos et al., 2025; Chen et al., 2021). Clinician and pharmacist feedback constitutes a central component of this bidirectional system. When healthcare providers interpret and respond to DDI alerts, they apply mechanistic understanding alongside patient-specific priorities, comorbidity burden, and therapeutic goals. Their decisions represent context-specific clinical reasoning that cannot be inferred from experimental data or mechanistic models alone representing valuable signals within a continuous validation and learning process (Haefner et al., 2021). These responses therefore constitute valuable input that can improve interpretive accuracy and strengthen alignment with clinical relevance. Comparable human-in-the-loop approaches have been applied in medical image analysis, where AI systems are iteratively developed, evaluated, and refined based on expert feedback and discussed considering pharmacovigilance tools (Lang et al., 2024; Raumviboonsuk et al., 2019; Shamim et al., 2024).

Methodological paradigms such as distributed and federated learning architectures provide feasible foundations for such adaptive systems while preserving data privacy (Nasajpour et al., 2025; Kim et al., 2025). Within such architectures, clinician feedback can function as weakly structured learning signals consistent with real-world CDSS workflows (Lampe et al., 2024). Heterogeneity or disagreement among clinicians reflects the inherent variability of biomedical evidence and can be addressed through reputation-aware aggregation strategies (Lampe et al., 2024; Cai et al., 2025). Validation of HAMEAI-aligned systems would therefore rely on established CDSS evaluation frameworks, prioritizing complementary outcomes, process, and harm-related metrics over single performance indicators. Considering operational parameters such as update frequency and relative services, they are left unspecified, as they depend on clinical context, regulatory constraints, and institutional capacity which fall beyond the scope of the current perspective.

In this paradigm, HAMEAI represents an approach in which AI does not operate autonomously but evolves in conjunction with human expertise (Figure 1). Clinician overrides are no longer interpreted as failures but as informed, patient-specific decisions that recalibrate risk thresholds and prioritize clinically meaningful signals. This approach supports identification of cumulative or clustered moderate-risk DDIs, early ADR signals, and patterns that might otherwise lead to prescribing cascades (Table 1). Explainability is intrinsic rather than auxiliary, enabling progressive refinement of pharmacological reasoning and safer prescribing across diverse populations.

**TABLE 1:** Illustrative examples of moderate DDIs that escalate to high-risk, clinically significant DDIs under contextual modifiers, as framed within the perspective of the HAMEAI framework.

Drug combination (moderate DDIs)	Mechanism (PK/PD)	Contextual risk factors	Potential Clinical impact	Risk of prescribing cascade	AI-prompt information (HAMEAI): HPR verification (validate and retain)
Atorvastatin + clarithromycin	PK: CYP3A4 inhibition	Age >65, hepatic impairment	Myopathy, rhabdomyolysis	Myalgia → NSAIDs or statin stop	CYP3A4 block → HPR verify age/LFTs
Metoprolol + salbutamol	PD: β1/β2 antagonism	Smoking, COPD/asthma, air pollution	Bronchospasm, cough	Cough misdiagnosed as infection → antibiotics	β-blocker–β-agonist conflict → HPR verify COPD/asthma and environment
Salbutamol + erythromycin	PD: QT prolongation	Elderly, hypokalemia, polypharmacy	Arrhythmia, torsades	Syncope → antiarrhythmic added	QT risk ↑ → HPR verify ECG/K+
Tramadol + paroxetine	PK/PD: CYP2D6 dependence	CYP2D6 IM, multimorbidity	Poor analgesia, ADRs	Dose escalation or opioid switch	Pharmacogenomics CYP2D6 effect → HPR verify genotype/response
Metoprolol + paroxetine	PK: CYP2D6 inhibition	CYP2D6 IM, older age	Bradycardia, hypotension	Falls → antihypertensive changes	Pharmacogenomics CYP2D6 inhibition → HPR verify HR/BP trend
Simvastatin + grapefruit	PK: Intestinal CYP3A4 inhibition	Daily intake, high dose, obesity	Myopathy, rhabdomyolysis	Muscle pain → analgesics	Diet–drug interaction → HPR verify grapefruit intake; inform/about diet habits, and confirm avoidance
Atorvastatin + St John’s wort	PK: CYP3A4 induction	OTC supplement use	Treatment failure	Dose increase or statin switch	Supplement–drug interaction → HPR verify use; inform about supplements and confirm avoidance
Mirtazapine + pregabalin	PD: Additive CNS depression	Age >65, frailty, renal impairment	Ataxia, cognitive decline, falls, bone fracture	Fracture → opioid added (codeine) Misdx dementia (donepezil) →Respiratory depression, delirium, confusion, bradycardia	CNS load ↑ → HPR verify gait/cognition/renal function. Re-assess and confirm
Apixaban + diltiazem + clarithromycin	PK: Additive inhibition	Polypharmacy, frailty	Toxic drug levels (apixaban) bleeding and need for hospitalization	Anemia meds (i.e., ferrous supplements)	Cumulative inhibition (CYP3A4; P-gp) → HPR confirm dose/renal function and follow-up
NSAID + ACE-I+ diuretic	PK/PD: Renal hypoperfusion	Age >65, dehydration	AKI and HF worsening	CKD misdx → chronic medications; intensification of HF-medications	“Triple whammy” alert. HPR HPR verify hydration/renal function
SSRI + ASA	PD: Platelet inhibition	Elderly, anticoagulation OTC risk	GI bleeding	PPI added → new DDIs	Platelet inhibition ↑ → HPR verify GI bleeding risk/anticoagulation

ACE-I: angiotensin converting enzyme inhibitors; AKI, acute kidney injury; ASA, acetylsalicylic acid; BP, blood pressure; CKD, chronic kidney disease; CL, clearance; COPD, chronic obstructive pulmonary disease; CYP, Cytochrome P450; GI, gastrointestinal; HPR, Healthcare Professional/Researcher; HR, heart rate; IM, intramuscular; LFT, liver function tests; misdx, Misdiagnosis; NSAIDs, Non-steroidal Anti-inflammatory Drugs; OTC, Over-the-counter; PD, pharmacodynamic; PK, pharmacokinetic; PPI, proton pump inhibitors; SSRI, selective serotonin reuptake inhibitors; ↑, Increase; →, Leads to).

But to do so, emerging frameworks of HAMEAIs should not only recognize vulnerabilities associated with pharmacological mechanisms but also incorporate individualized patterns of risk arising from SDoH and healthcare engagement including potential behavioral tendencies and psychosocial stressors, (i.e., due to an associated stigma with a treatment), nutritional influences, or environmental exposures (Campbell et al., 2023; Li H. et al., 2023; Kågström et al., 2025). The long-term goal is the development of a system that evolves in parallel with pharmacological science and clinical practice, supported by systematic data acquisition on high-risk DDIs and real-time clinical outcomes or scenarios (Ali et al., 2025). This evolution does not replace pharmacological reasoning; rather, it strengthens it by supplying healthcare providers (and in an extend researchers) with detailed, context-sensitive insights that anticipate potential DDIs before they manifest clinically and support individualized therapeutic adjustments based on risk stratification. Ultimately, integrating adaptive AI-DDI systems across both drug development and clinical practice could harmonize mechanistic predictions with the real-world behavior of medicinal products. Such systems hold promises for advancing regulatory science, informing early-stage compound screening, and elevating the precision and safety of everyday pharmacotherapy.

### Towards a transparent, patient-centered, and mechanistically aware AI ecosystem

Integrating bidirectional learning within an ethical, transparent, and clinically trustworthy framework is essential for advancing DDI management. A modern AI ecosystem must make pharmacology-driven reasoning visible and interpretable, avoiding the opacity that often accompanies complex computational approaches (Thalpage, 2023). Transparency is not merely a technical preference but a professional and ethical necessity: clinicians must understand why an interaction is flagged, which mechanistic pathways or PK/PD factors dominate the inference, and how patient-specific characteristics such as pharmacogenetic variants, comorbidities, or SDoH modulate predicted risk (Kierner et al., 2023). Despite their promise, these approaches face significant ethical and practical challenges, including dataset bias, incomplete electronic health records, the need for explainability in high-stakes decisions, and emerging regulatory demands for transparency and ongoing model updates (Mohsin Khan et al., 2025; Taddese et al., 2025; Pham, 2025). Adaptive human-in-the-loop systems also raise questions regarding data privacy, accountability, informed consent, and performance drift under continuous learning. While a full ethical analysis lies beyond the scope of this Perspective, HAMEAI aligns with principles of responsible AI by emphasizing clinician oversight, interpretability, and transparency rather than autonomous decision-making (Abdalla et al., 2025). Existing regulatory frameworks at the FDA and EMA, largely designed for static algorithms, struggle to accommodate lifecycle-based updates and real-world monitoring (Onitiu et al., 2024; Freyer et al., 2025; Zaidan and Ibrahim, 2024). Emerging policy scholarship increasingly advocates adaptive governance models capable of reconciling clinician-centered learning loops with evolving oversight structures (Ganna et al., 2024). In this context, HAMEAI is positioned as a conceptual research framework designed to catalyze innovation, rather than a system prepared for operational deployment within today’s regulatory restrictions (Sokol et al., 2025; Laranjo et al., 2025). Addressing these challenges requires a collaborative, multidisciplinary effort among healthcare professionals, similar to the distributed data collection and analysis seen in some citizen science projects (Jiwani et al., 2020; Downe et al., 2019).

Embedding mechanistic explainability in this context allows the identification of false positives, de-emphasis of DDIs that are clinically irrelevant, and prioritization of those risks that truly affect patient safety (Vo et al., 2022; Thalpage, 2023; Zhong et al., 2023; Wani et al., 2024; Carloni et al., 2025). Such clarity ensures that the clinician’s judgment remains central while data-driven insights augment decision-making. Ethical principles require that data integration respect patient privacy, prevent misuse, and maintain autonomy while enabling clinicians to provide evidence-based reasoning for DDI risk mitigation and preventive strategies (Pham, 2025; Hey and Kulkarni, 2025; Singh et al., 2025). A patient-centered orientation situates the AI system within the broader goal of improving therapeutic outcomes rather than solely optimizing computational performance. By integrating real-world observations with mechanistic and research data, adaptive AI-DDI systems can reveal previously unrecognized interaction pathways, clarify when presumed high-risk DDIs are unlikely to be clinically significant, reduce alert fatigue, and support proactive pharmacovigilance (Létinier et al., 2021; Praveen et al., 2023; Lee et al., 2023; Shamim et al., 2024; Mishra and Gupta, 2025).

In this envisioned ecosystem, AI strengthens rather than replaces clinical expertise, enhancing mechanistic understanding, patient-specific risk assessment, and the safety and precision of therapeutic decision-making. The resulting insights can also inform updates to product labeling, guide drug development, and anticipate interaction risks early in the R&D pipeline.

## Conclusion

Identifying DDIs in drug research and successful management in clinical practice requires integrating mechanistic pharmacology with the dynamic complexity of real-world patient care. Looking ahead, AI-DDI systems that integrate mechanistic clarity with patient-centered contextual awareness may fundamentally reshape how we interpret and prevent DDIs. They may lay the foundation for next-generation HAMEAI systems capable of integrating mechanistic clarity with real-world clinical data. Such systems promise not only better predictions but a deeper, continuously updated understanding of pharmacological behavior across heterogeneous patient populations.

## Data Availability

The original contributions presented in the study are included in the article/supplementary material, further inquiries can be directed to the corresponding authors.
